# Different Algicidal Modes of the Two Bacteria *Aeromonas bestiarum* HYD0802-MK36 and *Pseudomonas syringae* KACC10292^T^ against Harmful Cyanobacteria *Microcystis aeruginosa*

**DOI:** 10.3390/toxins14020128

**Published:** 2022-02-08

**Authors:** Bum Soo Park, Chong-Sung Park, Yuna Shin, Sungae Yoon, Myung-Soo Han, Yoon-Ho Kang

**Affiliations:** 1 Marine Ecosystem Research Center, Korea Institute of Ocean Science and Technology, Busan 49111, Korea; parkbs@kiost.ac.kr; 2 Water Environmental Engineering Research Division, National Institute of Environmental Research, Incheon 22689, Korea; maeruginosa@gmail.com; 3 Water Environment Research Department, National Institute of Environmental Research, Incheon 22689, Korea; marianshin@korea.kr (Y.S.); ysa123@korea.kr (S.Y.); 4 Department of Life Science, College of Natural Sciences, Hanyang University, Seoul 04763, Korea; hanms@hanyang.ac.kr

**Keywords:** *Microcystis aeruginosa*, algicidal bacteria, *Aeromonas bestiarum*, *Pseudomonas syringe*, algicidal modes

## Abstract

Blooms of harmful cyanobacteria *Microcystis aeruginosa* lead to an adverse effect on freshwater ecosystems, and thus extensive studies on the control of this cyanobacteria’s blooms have been conducted. Throughout this study, we have found that the two bacteria *Aeromonas bestiarum* HYD0802-MK36 and *Pseudomonas syringae* KACC10292^T^ are capable of killing *M. aeruginosa*. Interestingly, these two bacteria showed different algicidal modes. Based on an algicidal range test using 15 algal species (target and non-target species), HYD0802-MK36 specifically attacked only target cyanobacteria *M. aeruginosa*, whereas the algicidal activity of KACC10292^T^ appeared in a relatively broad algicidal range. HYD0802-MK36, as a direct attacker, killed *M. aeruginosa* cells when direct cell (bacterium)-to-cell (cyanobacteria) contact happens. KACC10292^T^, as an indirect attacker, released algicidal substance which is located in cytoplasm. Interestingly, algicidal activity of KACC10292^T^ was enhanced according to co-cultivation with the host cyanobacteria, suggesting that quantity of algicidal substance released from this bacterium might be increased via interaction with the host cyanobacteria.

## 1. Introduction

A cyanobacterial bloom of *Microcystis aeruginosa* is a major threat in a freshwater ecosystem, since it adversely affects the freshwater ecosystem, drinking water safety, and human health [1,2,3,4,5,6]. To reduce this, extensive studies have been consistently conducted to develop an effective method to control this cyanobacterial bloom. Consequently, the various methods for physical (e.g., clay and ultrasound), chemical (e.g., copper sulfate, naphtoquinone derivates, and hydrogen peroxide), and biological (e.g., algicidal bacteria, fungi, and protozoa) controls have been developed [7,8,9,10]. Compared to physical and chemical controls, biological control was recognized as environmentally friendly technique for controlling this cyanobacterial bloom [11]. Thus, numerous studies on algicidal bacteria have been conducted, since bacteria are capable of controlling cyanobacteria blooms without side effects on other organisms in freshwater [12]. At the laboratory level, there have been numerous research studies where algicidal bacteria have been directly utilized to target organisms, but there is a significant limitation to the use of these bacteria for eliminating cyanobacteria blooms in fields; for example, in an open environment, their algicidal activity was not sustained as much as that under small scale laboratory condition, since it is difficult for them to form a stable micro-ecosystem in fields [13,14]. To resolve this limitation, an alternative way to use a bacterial consortium mixed with multiple-species algicidal bacteria is newly suggested [15]; there were joint effects of various algicidal bacteria in a pilot scale experiment (ca. 312 L). Interestingly, a bacterial consortium is capable of enhancing bacterial activities via synergistic interaction among bacteria [16]. Given these findings, it is important to construct a bacterial consortium, showing stable and enhanced algicidal activities even in fields.

To develop the new combination of algicidal bacteria, resulting in synergistic algicidal activity, it is required to collect more diverse algicidal bacteria and find the most effective combination for killing target harmful cyanobacteria. Therefore, as a first step, we aimed to identify the new algicidal bacteria against *M. aeruginosa* and analyze the algicidal range and mode of these bacteria in this study.

## 2. Results

### 2.1. Phylogenetic Position of Bacterial Isolate HYD0802-MK36

**Figure 1:**
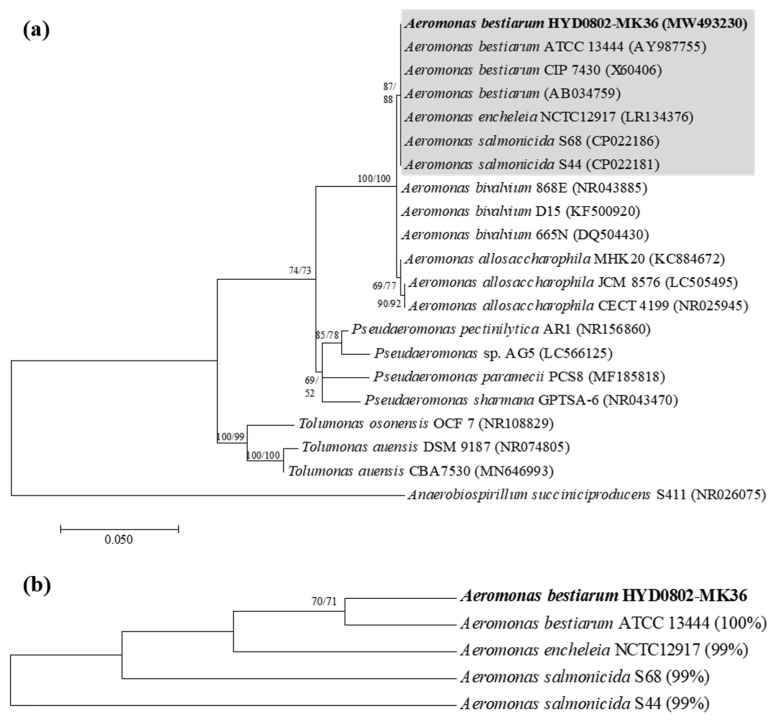
(**a**) A neighbor-joining (NJ) tree constructed with partial sequences of 16S rRNA gene of *Aeromonas* spp. and other relative bacteria *Pseudaeromonas* and *Tolumonas*. *Anaerobiospirillum succiniciproducens* S411 (NR026075) was used as the outgroup. (**b**) This tree was performed using only three species in the gray shaded box shown in Figure 1a. The numbers in parentheses indicated the sequence similarity compared to the HYD0802-MK36 sequence. An additional maximum-likelihood (ML) tree generated similar topology of the NJ, and thus its bootstrap values were incorporated into the NJ tree.

Phylogenetic position of HYD0802-MK36 that was newly isolated from the field throughout this study was determined based on partial sequences (1533 bp) of 16S ribosomal RNA (rRNA) (Figure 1). As a result, HYD0802-MK36 was grouped together with species of genus *Aeromonas*; *A. bestiarum* KTCC13444 (AY987755), *A. bestiarum* CIP7430 (X60406), *A. bestiarum* (AB034759), *A. encheleia* NCTC12917 (LR134376), and *A. salmonicida* S68 (CP022186), *A. salmonicida* S44 (CP022181) (Figure 1a). To identify this bacterial strain, additional phylogenetic analysis was carried out using the three *Aeromonas* species which showed ≥99% similarity (16S rRNA gene sequences) with HYD0802-MK36. Compared to other species, *A. bestiarum* was the phylogenetically closest species with HYD0802-MK36, and thus we identified HYD0802-MK36 as *A. bestiarum* (Figure 1b).

### 2.2. Algicidal Range of the Two Algicidal Bacteria

A total of 15 algal species (target and non-target species) were used to examine the algicidal range of the two bacterial strains; *A. bestiarum* HYD0802-MK36 and *P. syringae* KACC10292^T^ (Table 1). In this study, a bacterial strain which showed above 80% algicidal activity was defined to have a clear algicidal effect. Both bacterial strains showed a clear algicidal effect against target cyanobacteria *M. aeruginosa*; algicidal activities of HYD0802-MK36 and KACC10292^T^ were 91% and 96%, respectively. However, there was difference in algicidal range between the two bacterial strains. Algicidal activities (−143% to 20%) of HYD0802-MK36 against non-target algal species were generally lower than those of KACC10292^T^ (−47% to 83%), and KACC10292^T^ showed a clear algicidal effect (83%) on marine dinoflagellate *Akashiwo sanguinea*.

### 2.3. Algicidal Effect of the Two Bacteria on Microcystis aeruginosa

**Figure 2:**
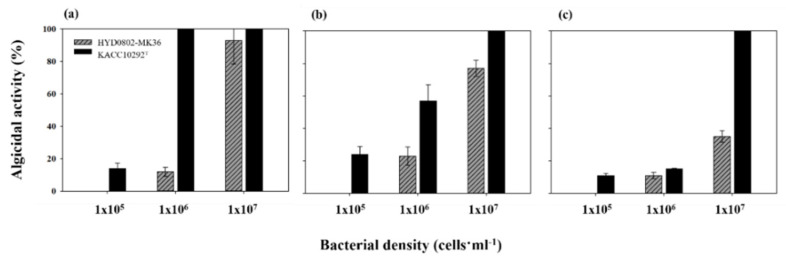
Algicidal activity (%) of inoculate bacterial densities of HYD0802-MK36 and KACC10292^T^ in various *M. aeruginosa* cell densities (**a**) 5 × 10^4^ (**b**) 5 × 10^5^ (**c**) 5 × 10^6^ cells mL^−1^. Bacterial density of each treatment indicated the average cell densities of cell counts (*n* = 10). The error bars represent the standard deviations.

To elucidate an optimum condition for algicidal activities of the two bacterial strains on target cyanobacteria, we inoculated the three different bacterial densities (10^5^, 10^6^, 10^7^ cells mL^−1^) into the three different densities (5 × 10^4^, 5 × 10^5^, 5 × 10^6^ cells mL^−1^) of *M. aeruginosa*, and their algicidal activity was measured. Algicidal activity was clearly varied according to both bacterial inoculation density and *M. aeruginosa* density; it was significantly elevated with higher inoculation density of bacteria and lower *M. aeruginosa* density (*p* < 0.01) (Figure 2). Algicidal activity of HYD0802-MK36 on *M. aeruginosa* was not clear (lower than 80%) when bacterial inoculation density was lower than 1 × 10^7^ cells mL^−1^, and it was gradually decreased according to increase in initial cell densities of *M. aeruginosa*; algicidal activity of this bacteria (inoculation density, 1 × 10^7^ cells mL^−1^) on 5 × 10^4^, 5 × 10^5^, 5 × 10^6^ cells mL^−1^ of *M. aeruginosa* were 93.0 ± 7.0, 77.6 ± 5.5, 35.4 ± 4.1%, respectively. Algicidal activity of KACC10292^T^ was generally higher than that of HYD0802-MK36; this bacterium completely eliminated target cyanobacterial cells, irrespective of cell densities of *M. aeruginosa*. In addition, unlike HYD0802-MK36, 100% of algicidal activity was shown when KACC10292^T^ was inoculated as 10^6^ cells mL^−1^ into 5 × 10^4^ cells mL^−1^ of *M. aeruginosa*.

### 2.4. Difference in Algicidal Modes of the Two Bacteria against Microcystis aeruginosa

**Figure 3:**
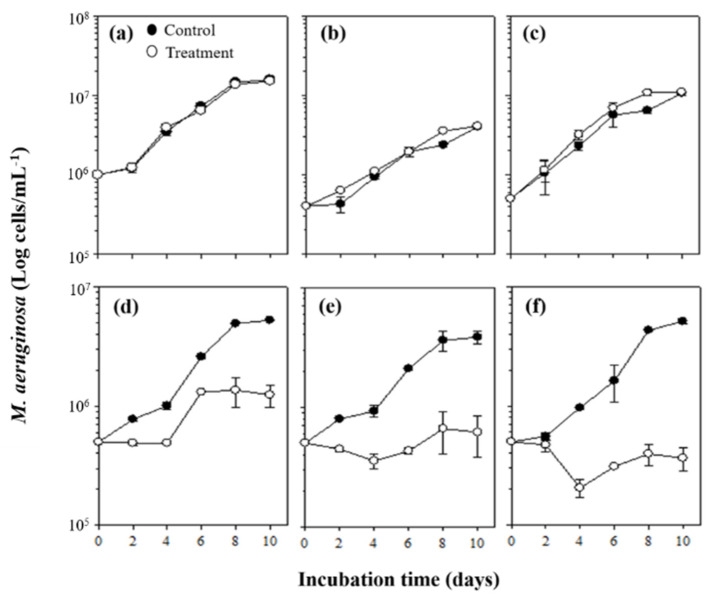
Algicidal activities of filtrates from co-culture of *M. aeruginosa* with the two bacterial strains. The ‘Control’ was *M. aeruginosa* incubated for 10 days in a solution obtained by sterilizing the co-culture of *M. aeruginosa* and bacteria using an autoclave. (**a**–**c**) In this treatment, *M. aeruginosa* was cultured in the filtrate of co-culture of *M. aeruginosa* and bacterium (HYD0802-MK36) with final concentrations (**a**) 10% (**b**) 50% (**c**) 90%, respectively. (**d**–**f**) *M. aeruginosa* incubated in the filtrate of co-culture of *M. aeruginosa* and bacterium (KACC10292^T^) with final concentrations (**d**) 10%, (**e**) 50%, (**f**) 90%.

To elucidate the algicidal mode of the two bacterial strains against target cyanobacteria, co-culture filtrates of *M. aeruginosa* and bacterial strains were inoculated into 10^6^ cells mL^−1^ of *M. aeruginosa* with the three different final concentrations (10, 50, and 90%), and algicidal activities of those filtrates were measured. It was observed that algicidal modes between the two bacterial strains against *M. aeruginosa* were completely different. The co-culture filtrate of HYD0802-MK36, irrespective of final concentrations, did not show algicidal activities against *M. aeruginosa* (Figure 3a,b). Whereas, co-culture filtrates of KACC10292^T^ clearly inhibited the growth of *M. aeruginosa* from 2 days after the addition of these filtrates, and the level of algicidal activity was gradually elevated according to increase in a final concentration with a high significance (*p* < 0.01) (Figure 3d–f); algicidal activities of 10, 50, and 90% co-culture filtrates of KACC10292^T^ were 78.6, 87.5, and 97.5%, respectively.

### 2.5. Subcellular Location of Algicidal Substances

To investigate the location of relevant algicidal substance(s) from the two bacterial strains, we measured the total proteins and the specific activities of mono- and co-culture filtrates and the cell-free extracts (total cell) (Table 2). Protein concentrations were commonly highest at the cell-free extract (57.9 and 53.8 mg L^−1^) in both HYD0802-MK36 and KACC10292^T^. However, the highest specific activity (=algicidal activity per protein concentration) of the two bacterial strains appeared at different fractions; cell-free extract (HYD0802-MK36, 372.3 units mg^−1^) and co-culture filtrate (KACC10292^T^, 11,635.8 units mg^−1^) were the fractions that showed the highest specific activities.

To fine-tune the subcellular location of algicidal substance(s), we subsequently examined the lysed host algal cells and obtained the three cell fractions; periplasm, cytoplasmic membrane, and cytoplasm. In the case of HYD0802-MK36, the highest specific activity was observed at cytoplasm fraction (60 mg L^−1^), but those of other fractions were relatively low (−6.3 to 4.4 mg L^−1^). In the case of KACC10292^T^, the cytoplasm exhibited higher specific activity (2227.5 units mg^−1^) than those of the periplasm (36.3 units mg^−1^) and cytoplasmic membrane (182.9 units mg^−1^) fractions, even though protein concentration was the lowest (1.0 mg L^−1^) in the cytoplasm when compared with the other fractions (periplasm: 18.1 mg L^−1^ and cytoplasmic membrane: 4.1 mg L^−1^).

## 3. Discussions

Throughout this study, we newly identified the two algicidal bacteria *A. bestiarum* HYD0802-MK36 and *P. syringae* KACC10292^T^ against harmful *M. aeruginosa* (Figure 1 and Figure 2). It is not surprising that these bacterial taxa show algicidal activities against this cyanobacteria *M. aeruginosa*. Based on previous studies, a number of bacterial species belonging to genera *Aeromonas* and *Pseudomonas* are capable of inhibiting the growth of cyanobacteria, including *M. aeruginosa* [17,18].

Algicidal activity against target cyanobacteria would be enhanced following an increase in inoculated bacterial densities, if the bacteria were responsible for algicidal activity. Based on our findings, this shows that algicidal activity originated from the inoculated bacteria.

The bacterial inoculum density [19,20,21] and physiological status (=growth phase) of host algae [4,22] are recognized as key factors, determining level of algicidal activity of bacteria. In our results, levels of algicidal activities of both bacterial strains against *M. aeruginosa* gradually elevated according to increase in inoculated density of each bacterial species (Figure 2). In addition, the two bacteria showed higher algicidal activity against lower cell densities (10^4^, 10^5^ cells mL^−1^) of *M. aeruginosa* than 10^6^ cells mL^−1^ of *M. aeruginosa*. Based on Mange et al. [20], algicidal bacteria are capable of more easily lysing host algal cells at the lag and exponential phases, compared to the stationary phase, due to the relatively weak cell wall, a result of active cell division. This might lead to reduction in level of algicidal activity of bacteria against the highest cell density (10^6^ cells mL^−1^) of *M. aeruginosa*, corresponding to the stationary phase.

There was a difference in the algicidal range of the two bacteria (Table 1). HYD0802-MK36 specifically attacked only target cyanobacteria *M. aeruginosa*, whereas the algicidal activity of KACC10292^T^ appeared as a relatively broad algicidal range in the following order of sensitivity: *M. aeruginosa* > *A*. *sanguinea* > *Scenedesmus acutus* > *Cyclostephanodiscus dubius*. Koss and Snyder [23] have classified biocontrol agents, including algicidal bacteria, into two groups: specialists and generalists. A specialist which has a tight dynamical linkage to their target (or host) species can selectively inhibit the growth of target species in a species-specific manner [24,25,26,27,28], whereas a generalist lacks target species specificity. According to this study, HYD0802-MK36 and KACC10292^T^ are likely to belong to the specialist and generalist categories, respectively. Interestingly, specialists are paid more attention, because of their advantages. The specialist algicidal bacteria facilitates the selective controlling of blooms of target species without disturbing non-target organisms [19], but a generalist algicidal bacteria may lead to unexpected adverse effect on non-target organisms [29,30].

In general, there are the two algicidal modes of bacteria, direct and indirect [19,21,31]. Based on our findings, the algicidal modes of the two bacteria were completely distinct (Figure 3). Unlike the direct addition of HYD0802-MK36 bacterial cells, algicidal activity disappeared when the filtrates of this bacteria were inoculated into *M. aeruginosa*. Moreover, the filtrates from mono- (bacteria only) and co-culture (bacteria and *M. aeruginosa*) similarly showed very low levels of algicidal activity (≤20 units mg^−1^) (Table 2). Contrarily, cell-free extract showed the highest algicidal activity (specific activity, 372.3 units mg^−1^). Given these findings, HYD0802-MK36 is able to kill *M. aeruginosa* cells only in case of direct cell-to-cell contact. We have observed the disappearance of algicidal activity in filtrates and cell-free extracts (including cell fractions) treated by heat and Proteinase-K (data not shown), suggesting that substances showing alga-lytic effects might be enzymes (Table 1). Interestingly, in previous studies [4,32], specific activity (unit mg^−1^) of a certain fraction of a bacterial cell was generally higher than that of cell-free extracts, but, in this study, specific activity of cell-free extract fraction was 6.4 times higher than the sum of specific activities of periplasm, cytoplasmic membrane, and cytoplasm. Taken together, this might indicate that a synergistic effect could occur due to the incorporation of enzymes in each bacterial cell fraction. However, due to a lack of evidence, it is impossible to fully elucidate the algicidal mechanism of HYD0802-MK3, and thus further studies are highly necessary.

Unlike HYD0802-MK36, the filtrates of KACC10292^T^ displayed a similar algicidal activity (≥78.6%) to direct addition of bacterial cells. Additionally, to identify characteristics of algicidal substance(s), we investigated the change in algicidal activity of the filtrates of this bacterium depending on heat (121 °C, 30 min) or Proteinase-K treatments. As a result, the algicidal activities of the filtrates commonly vanished after treatment with heat or Proteinase-K (data not shown). Given these findings, KACC10292^T^ can release certain substance(s), resulting in the cell death of *M. aeruginosa*, and this substance(s) is likely to be a protein, such as an enzyme, which has weak heat stability. However, in this study, we could not clearly identify the substances responsible for the cell lysis of *M. aeruginosa*. Therefore, further studies on identification of algicidal substance(s) are necessary. Interestingly, the specific activity level of co-culture filtrate (KACC10292^T^ and *M. aeruginosa*) was 36 time higher than that of mono-culture filtrate (Table 2). This shows that the concentration of algicidal substance released from bacterial cells were remarkably elevated in response to presence of target cyanobacteria *M. aeruginosa*. Together with these findings, there might be a strong interaction between KACC10292^T^ and *M. aeruginosa*; for example, this bacterium can recognize *M. aeruginosa* cells, and it actively releases algicidal substance(s) in order to kill this cyanobacterium.

## 4. Conclusions

Throughout this study, we newly found the two bacteria with algicidal activity against harmful cyanobacteria *M. aeruginosa*. These two bacteria were completely different in an algicidal mode. HYD0802-MK36, as a direct attacker, can lyse *M. aeruginosa* cells using enzymes when direct cell (bacteria)-to-cell (cyanobacteria) contact happens, whereas KACC10292^T^, as an indirect attacker, releases an algicidal substance (e.g., enzyme) which is mainly located in cytoplasm, irrespective of the presence of *M. aeruginosa*. Interestingly, the quantity of algicidal substance released from this bacterium may be increased when it co-cultivates with the host cyanobacteria. Together with these findings, these two new algicidal bacteria can be useful bio-resources for controlling blooms of *M. aeruginosa* in freshwater. Based on a recent study [15], a bacterial consortium mixed with multiple-species algicidal bacteria is suggested as an alternative to resolve a limitation (e.g., unstable and lowered algicidal effect in fields), and thus it is thought to be important to find a bacterial consortium, showing stable and effective algicidal activities against *M. aeruginosa* even in fields. Besides, it is unclear whether or not synergistic effect can be shown in case a bacterial consortium is formed with the combination of bacterial strains which have distinct algicidal modes. Therefore, further studies will be conducted to find the most effective combination of algicidal bacteria, including HYD0802-MK36 and KACC10292^T^.

## 5. Materials and Methods

### 5.1. Algal Cultures

Algal cultures were obtained from the Han laboratory, Hanyang University (HY, Korea), the Korean Marine Microalgae culture (KMMCC, Korea), the National Institute for Environmental Studies (NIES, Japan), and the University of Toronto Culture Collection of Algae and Cyanobacteria (UTCC, Canada) (Table 1). The cultures were incubated at different conditions depending on algal taxa; (i) green algae and cyanobacteria were incubated at 25 ℃ in CB [33] medium under cool-white fluorescent lamps (50 μmol photons ms^−2^ s^−1^) on a 12:12 (light:dark); (ii) diatom, dinoflagellate, and Raphidophyte were maintained in diatom medium (15 °C) [34] and F2-Si medium (20 ℃, 32 psu) [35] under the same light conditions.

### 5.2. Isolation and Screening of Algicidal Bacteria

To directly isolate algicidal bacteria against *M. aeruginosa*, the samples were collected from the water and wet soil samples at the Daewang Reservoir (87 sites) located in Seongnam-si, Gyeongi-do, Korea when blooms of *M*. *aeruginosa* occurred in 2008, and bacterial strains were isolated using the samples according to the modified method described in Walker and Patrick [36]. Sterile sodium alginate (2% *w*/*v*) in distilled water was mixed with *M. aeruginosa* cultures at the ratio of 1:1. The mixture was dripped into sterile 0.25 M calcium chloride to produce gel beads of calcium alginate. The gel beads were incubated into 50 mL of the filtered (0.22 μm pore diameter) natural samples for 3 days under cyanobacterial growth condition, and they were transferred into 25 mL of Nutrient Broth (NB) medium for 2 days incubation at 25 ℃ under shaking condition (200 rpm). Then, 0.1 mL was taken from this NB medium after 2 days incubation, and it was incubated onto NB agar plate (25 ℃, dark condition) to isolate a single colony. Each bacterial isolate was incubated in 5 mL of NB medium under the same incubation condition for 1–2 days. The resultant bacterium was harvested by centrifugation at 10,000× *g* for 10 min, and adjusted to an OD_660_ of 1.5 with CB medium (yielding an initial density of ca 2 × 10^8^ cells mL^−1^). The density of bacterial cells was determined by 4’, 6-diadimino-2-phenylindole (DAPI) staining [37] as follows: bacterial cells were fixed with 2% glutaraldehyde (final concentration) and filtered onto black polycarbonate filters (0.22 μm pore diameter), and cell numbers were counted at a magnification of ×400 using an epifluorescence microscope (Carl Zeiss, Jena, Germany). To screen algicidal bacteria, each bacterial strain was inoculated into *M. aeruginosa* cultures at a final concentration of ca. 1 × 10^5^ cells mL^−1^. After 10 days’ incubation, *M. aeruginosa* cells were counted using haemocytometer (Superior Marienfeld, Lauda-Konigshofen, Germany) under microscopic observation at a magnification of ×100 to ×400, and we searched algicidal bacteria, showing 90% of algicidal activity against *M. aeruginosa*. Additionally, for the screening of another algicidal bacterium, 26 strains belonging to genus *Pseudomonas* were obtained from the Korean Agricultural Culture Collection (KACC), and algicidal activities of these bacterial strains were tested to screen algicidal bacteria using the same method above. In this manner, we successfully isolated the two strains HYD0802-MK36 and KACC10292^T^ which showed algicidal activities against *M. aeruginosa*.

### 5.3. Bacterial Identification

To identify the bacteria strain HYD0802-MK36, its genomic DNA was isolated as previously described [4], and was identified by phylogenetic analysis. Polymerase chain reaction (PCR) was performed using an i-cycler (Bio-Rad, Hercules, CA, USA) with two universal primers, 27F (5′-AGAGTTTGATCATGGCTCAG-3′) and 1492R (5′-GGTTACCTTGTTACGACTT-3′) [38] in a 50 μL reaction mixture that contained 20 ng template DNA, 1× PCR Buffer (Mg^2+^ Free), 5 mM MgCl_2_, 10 mM dNTP, 10 pM of each primer, and 2.5 units of taq DNA polymerase. The PCR was run with 35 thermal cycles of denaturation for 1 min at 95 ℃, annealing for 1 min at 55 ℃, extension for 1.5 min at 72 ℃, and with a final elongation step of 10 min at 72 ℃.

Phylogenetic analyses were performed by considering the sequences of our isolate and the related sequences retrieved from public database. They were aligned using the Sequencher (version 5.1, Gene Codes Corp., Ann Arbor, MI, USA, 2012). The alignment was edited manually. We determined partial sequences of the 16S rRNA (1467 bp). However, it was difficult to identify the species level with the available length of this nucleotide sequence. Therefore, phylogenetic analysis was performed by considering a longer aligned sequence (1533 bp) with certain related sequences. Sequences were deposited in GenBank (http://www.ncbi.nlm.nih.gov (accessed on 28 February 2021)) with accession number (MW493230). The topology for the phylogenetic tree was derived using the maximum likelihood (ML) analysis. For this analysis, the GTR+G+I (the General Time Reversible model incorporating in variant sites and a gamma distribution) model was selected as the best model at MEGA 7.0 [39]. The MEGA 7.0 software was used to ML, and neighbor-joining (NJ) analysis using the *p*-distance models. All analyses were tested using bootstrapping with 1000 replicates.

### 5.4. Algicidal Range of the Two Bacteria

We investigated the host range of algicidal bacteria on 15 algae and cyanobacterial abundance in single-species tests (Table 1). Each bacterial culture (0.5 mL, 2 × 10^8^ cells mL^−1^) was inoculated into 6-well plate containing 4.5 mL of various host strains. The 15 species considered were Diatom (*C*. *dubius*, *C*. *meneghiniana*, *S*. *hantzschii*), Green algae (*C*. *cambricum*, *Pediastrum* sp., *S*. *acutus*), Cyanobacteria (*M*. *aeruginosa*), Dinoflagellates (*A*. *sanguinea*, *A*. *catenella*, *H*. *triquetra*, *P*. *micans*), and Rapidophyte (*C*. *marina*, *C*. *ovata*, *F*. *japonica*, *H*. *akashiwo*). These algae and cyanobacteria were incubated for 10 days under the aforementioned growth conditions. The cell densities of algae and cyanobacteria were counted at Day 10 (after addition of bacteria), and the algicidal activity (%) was calculate following the equation as below (the Section 5.8).

### 5.5. Algicidal Activity at Different Bacterial Densities

To elucidate algicidal activity at different initial densities of the two bacteria (HYD0802-MK36 and KACC10292^T^) against *M. aeruginosa*, 0.5 mL aliquots of serially diluted bacterial samples (1 × 10^5^, 1 × 10^6^, 1 × 10^7^ cells mL^−1^) were inoculated into 4.5 mL of serially diluted *M. aeruginosa* culture (5 × 10^4^, 5 × 10^5^, 5 × 10^6^ cells mL^−1^), respectively. Then, we counted cell densities of *M. aeruginosa* at Day 2, 4, 6, 8, and 10, and calculated the algicidal activity (%) following the equation as written below (the Section 5.8).

### 5.6. Determination of Algicidal Modes

We determined whether *M. aeruginosa* and algicidal bacteria required direct contact for their algicidal action. Each bacterial type (0.5 mL, 2 × 10^8^ cells mL^−1^) were added to 4.5 mL of *M. aeruginosa* strain (1.1 × 10^5^ cells mL^−1^, in exponential phase) in a 6-well plate. After 90% or more algal cells were lysed, *M. aeruginosa* culture-inoculated bacteria was filtered through 0.2 µm membrane filter, and 0.5, 2.5, and 4.5 mL of the resultant filtrate were individually inoculated into 6-well plate containing 4.5, 2.5, and 0.5 mL *M. aeruginosa* strains (1.1 × 10^5^ cells mL^−1^, in exponential phase). The final proportion of bacteria and algae was 10%, 50%, and 90%, respectively. Subsequently, the number of *M. aeruginosa* cells in each experimental group was counted every 2 days.

### 5.7. Algicidal Activities of Cell Fractions of the Two Bacteria

We referred to Kang et al. [4] for the preparation of cell-free extracts and culture supernatants. To obtain cell fractions, each bacterial pellet was suspended in phosphate buffer (pH 9) containing 50 mM EDTA and incubated at 40 ℃ for 30 min. The periplasm fraction was obtained by centrifuging at 17,000× *g* for 30 min at 4 ℃. Additionally, to obtain the cytoplasm and cytoplasmic membrane, the spheroplast without cell wall was homogenized using a mortar and pestle for 90 min in 10 mM phosphate buffer (pH 7) at 5 ℃ and centrifuged at 21,000× *g*. The supernatant of cytoplasm fraction was separated by centrifugation (Tomy Seiko Ltd., Tokyo, Japan) at 90,000× *g* for 5 h at 4 ℃. The pellet was resuspended in 10 mM phosphate buffer (pH 7) and used as the cytoplasmic membrane fraction [40].

We quantified the protein content of all fractions and culture supernatants according to the Bradford method [41] with bovine serum albumin (Amersham-Pharmacia Biotech, Uppsala, Sweden) as the protein standard. *M. aeruginosa* was treated with various protein fractions (20% *v*/*v*) in 24-well plates (Falcon, Lincoln Park, NJ, USA), and the cyanobacterial cells were counted after 24 h as above. A unit of enzyme is defined as the amount of enzyme that reduced 10^4^ cells after 24 h.

### 5.8. Data Analysis

All experiments were repeated in triplicate and the results are provided by the mean and standard deviation of the raw data. The algicidal activity (%) of each algal species by algicidal bacteria was calculated by the following equation: Algicidal activity (%) = (1 − *Tt*/*Ct*) × 100, where *T* (treatment) and *C* (control) are the algal cell densities with- and without algicidal bacteria, respectively, and *t* is the inoculation day. Analysis of covariance (ANCOVA) was used to determine if the differences were statistically significant (*p* < 0.05) through time in responses between control and treatment. All statistical analyses were performed using the SPSS software (version 17.0, SPSS Inc., Chicago, IL, USA, 2019).

## Figures and Tables

**Table 1:** Algicidal range of the two bacterial against target and non-target algal species.

Algal Species	Strain Number	Algicidal Activity (%)	
		MK36	KACC10292^T^
*Coelastrum cambricum*	HYS0706-C3	11	0
*Cyclostephanodiscus dubius*	HYN0410-A4	0	57
*Cyclotella meneghiniana*	HYK0210-A1	−143	39
*Microcystis aeruginosa*	NIES-298	91	96
*Pediastrum* sp.	HYY0901-A18	−21	27
*Scenedesmus acutus*	NIES-94	20	68
*Stephanodiscus hantzschii*	UTCC267	−38	45
*Akashiwo sanguinea*	HYCW-A20	−20	83
*Alexandrium catenella*	HYCW-A21	−15	62
*Chattonella marina*	HYCW-A22	−20	10
*Chattonella ovata*	HYCW-A23	−15	15
*Fibrocapsa japonica*	KMMCC133	−20	25
*Heterocapsa triquetra*	HYCW-A24	0	−20
*Heterosigma akashiwo*	HYCW-A25	−11	−47
*Prorocentrum micans*	HYCW-A26	−20	−20

**Table 2:** Degradation of *M. aeruginosa* NIES 298 by cell culture filtrates, cell-free extract of isolate algicidal bacteria and various fractions of cell-free extracts of isolate algicidal bacteria. *^a^* Monoculture filtrate of bacterial strain in CB medium; *^b^* co-culture filtrate of bacterial strain with *M. aeruginosa*; *^c^* the specific activity was calculated as follows: specific activity (units mg^−1^) = Total activity total protein^−1^.

Fraction	Protein Concentration (mg L^−1^)		Total Activity (Units)		Specific Activity (Units mg^−1^) *^c^*	
	MK36	KACC10292^T^	MK36	KACC10292^T^	MK36	KACC10292^T^
Monoculture filtrate *^a^*	0.4	1.2	0.0	31.7	0.0	322.3
Mixed-culture filtrate *^b^*	24.0	1.7	480.0	19,780.9	20.0	11,635.8
Cell-free extract	57.9	53.8	21,556.0	20,430.0	372.3	379.7
Periplasm	9.0	18.1	39.5	658.5	4.4	36.3
Cytoplasmic membrane	5.6	4.1	-134.0	750.0	-6.3	182.9
Cytoplasm	21.3	1.0	336.0	2227.5	60.0	2227.5

## Data Availability

Not applicable.
